# Perspectives and experiences of patients and healthcare professionals with geriatric assessment in chronic kidney disease: a qualitative study

**DOI:** 10.1186/s12882-020-02206-9

**Published:** 2021-01-06

**Authors:** Carlijn G. N. Voorend, Noeleen C. Berkhout-Byrne, Yvette Meuleman, Simon P. Mooijaart, Willem Jan W. Bos, Marjolijn van Buren

**Affiliations:** 1 Department of Internal Medicine (Nephrology), Leiden University Medical Centre, Albinusdreef 2, 2333 ZA Leiden, The Netherlands; 2 Department of Clinical Epidemiology, Leiden University Medical Centre, Leiden, The Netherlands; 3 Department of Gerontology and Geriatrics, Leiden University Medical Centre, Leiden, The Netherlands; 4 Department of Internal Medicine, St. Antonius Hospital, Nieuwegein, The Netherlands; 5 Department of Nephrology, Haga Hospital, The Hague, The Netherlands

**Keywords:** Qualitative research, Focus groups, Frail older adults, Geriatric assessment, Chronic kidney failure, ESRD, Decision making, shared, Quality of life

## Abstract

**Background:**

Older patients with end-stage kidney disease (ESKD) often live with unidentified frailty and multimorbidity. Despite guideline recommendations, geriatric assessment is not part of standard clinical care, resulting in a missed opportunity to enhance (clinical) outcomes including quality of life in these patients. To develop routine geriatric assessment programs for patients approaching ESKD, it is crucial to understand patients’ and professionals’ experiences with and perspectives about the benefits, facilitators and barriers for geriatric assessment.

**Methods:**

In this qualitative study, semi-structured focus group discussions were conducted with ESKD patients, caregivers and professionals. Participants were purposively sampled from three Dutch hospital-based study- and routine care initiatives involving geriatric assessment for (pre-)ESKD care. Transcripts were analysed inductively using thematic analysis.

**Results:**

In six focus-groups, participants (*n* = 47) demonstrated four major themes: *(1) Perceived characteristics of the older (pre)ESKD patient group*. Patients and professionals recognized increased vulnerability and (cognitive) comorbidity, which is often unrelated to calendar age. Both believed that often patients are in need of additional support in various geriatric domains. *(2) Experiences with geriatric assessment.* Patients regarded the content and the time spent on the geriatric assessment predominantly positive. Professionals emphasized that assessment creates awareness among the whole treatment team for cognitive and social problems, shifting the focus from mainly somatic to multidimensional problems. Outcomes of geriatric assessment were observed to enhance a dialogue on suitability of treatment options, (re)adjust treatment and provide/seek additional (social) support. *(3) Barriers and facilitators for implementation of geriatric assessment in routine care.* Discussed barriers included lack of communication about goals and interpretation of geriatric assessment, burden for patients, illiteracy, and organizational aspects. Major facilitators are good multidisciplinary cooperation, involvement of geriatrics and multidisciplinary team meetings. *(4) Desired characteristics of a suitable geriatric assessment* concerned the scope and use of tests and timing of assessment.

**Conclusions:**

Patients and professionals were positive about using geriatric assessment in routine nephrology care. Implementation seems achievable, once barriers are overcome and facilitators are endorsed. Geriatric assessment in routine care appears promising to improve (clinical) outcomes in patients approaching ESKD.

**Supplementary Information:**

The online version contains supplementary material available at 10.1186/s12882-020-02206-9.

## Background

In older patients with end stage kidney disease (ESKD) frailty, malnutrition, cognitive impairments, depression and impaired health-related quality of life are highly prevalent [1–3] and often underdiagnosed [4, 5]. Geriatric impairments are strongly associated with adverse health outcomes such as hospitalisation and mortality in (pre-)ESKD patients [3, 6]. Recently published ESKD guidelines recommend to frequently measure different geriatric domains and to use these outcomes in the decision making process [7, 8], but this is not part of standard clinical care [9].

The gold standard to geriatric appraisal is the Comprehensive Geriatric Assessment (CGA); a multidisciplinary process to identify older patients’ medical, psychosocial and functional needs, and to develop a coordinated integrated care plan [10]. CGA has been shown to improve outcomes including hospitalisation and mortality, and may potentially also reduce costs [11, 12]. However, for implementation in standard clinical care, full CGA often poses logistical difficulties [10]. Contrarily, short frailty screening tools are insufficiently discriminative for clinical use in ESKD patients [13]. In medical areas such as oncology, acute hospital settings and perioperative care, new models and settings of geriatric assessment are being explored [10], but limited attempts in nephrology are reported [4, 14–17]. So far, no standardized approach exists and thus, the search for the optimum use of geriatric assessment in routine care of older patients approaching ESKD continues [9].

To develop a nephrology-tailored routine geriatric assessment (NGA), it is crucial to understand patients’ and professionals’ perspectives on current practices of geriatric assessment in nephrological care. Until now, this has not yet been properly investigated [14]. Qualitative research can provide valuable insights into stakeholders’ perceived effectiveness of care pathways [18]. The primary aim was to elicit perspectives and experiences of patients and professionals with geriatric assessment in the care for older (≥65 years) patients approaching ESKD (estimated glomerular filtration rate < 20 ml/min/1.73m^2^), and to identify benefits, facilitators and barriers for implementation into routine nephrological care.

## Methods

### Study design and participants

We conducted a qualitative study, approved by the Medical Research Ethics Committee United (MEC-U, Nieuwegein, The Netherlands, reference W17.127), using focus group discussions. The study population comprised of (pre)ESKD patients, caregivers and healthcare professionals who were experienced with hospital-based geriatric assessment practices in nephrology. The three different practices included (i) a yearly one-hour geriatric assessment in routine care for patients approaching ESKD performed in a University hospital, (ii) a three-hour geriatric assessment for patients approaching ESKD in a study setting, of which results were used in routine-care [15], and (iii) a single-time point geriatric assessment among patients starting with or withholding from dialysis for study purposes, to be measured in a home-visit [4]. Table 1 shows detailed information on the geriatric assessment practices, population of interest, and different instruments that were used. For each of the three assessment practices we initially planned one focus group for the patient-perspective (including caregivers) and one professional group.

**Table 1:** Overview of geriatric assessment practices, from which participants were recruited, including geriatric testing methods

Practices	COPE study [15]	GOLD study [4]^b^	Routine care pathway, University Medical Centre Groningen
Focus group number	1, 2	3, 4	5, 6
Type of practice	Prospective multicentre observational cohort study 4 year follow up	Prospective multicentre observational cohort study (cross-sectional geriatric assessment)	Routine care practice
Aim of geriatric assessment	Examine the severity of cognitive impairment in older patients reaching ESKD before dialysis and the rate of decline after dialysis or CCM initiation	Assess the association of geriatric measures between start of dialysis and after 6 months	Guide patients to the best treatment choice and to define supplementary care to optimize quality of life and reduction of illness-related symptoms
Population at inclusion	≥65 years, eGFR ≤20 ml/min/1.73m^2^	≥65 years, initiating dialysis or conservative kidney management	≥70 years (or younger if indicated), eGFR ≤20 ml/min/1.73m^2^
Measurements	Baseline: at inclusion Follow-up: yearly (four times), and after six months of start dialysis treatment	Baseline: within 4 weeks of initiating dialysis or 4 weeks after final decision to withhold Follow-up: after 6 months by phone	Yearly assessment divided over 2 or 3 visits
Conducted by	Nurse practitioner or geriatric nurse	Research nurse	Nurse practitioner
Duration	3 h	60–90 min	2 × 30 min
Location	Outpatient clinic	Home visit, follow-up by telephone	Outpatient clinic
Use of outcomes of assessment	For study purposes and discussed in multidisciplinary meeting and with patient, if necessary referred for geriatric consult	Collected for study purposes only, at home or in dialysis centre	Discussed in multidisciplinary meeting and with patient
**Geriatric measures:**			
1. Functional status (ADL/ iADL)	GARS^a^ Lawton^a^	Katz-6^a^ Lawton^a^	Katz^a^
2. Mobility	Gait speed^a^ Hand grip strength^a^ Short Physical Performance battery^a^	Timed up and go Falls^a^ Four Test Balance Scale	Timed up and go^c^ Falls^c^
3. Cognition	Mini Mental State Examination^a^ Clock drawing^a^ 15- WVLT^a^ Stroop Colour Word Test^a^ Trail making test (A&B)^a^ Visual Association Test^a^ Letter Digit Substitution Test^a^ Assessment of numeracy^a^	Mini Mental State Examination Clock drawing Enhanced Cued Recall Semantic Fluency Test	MOCA^a^
4. Mood	Geriatric Depression Scale^a^	Geriatric Depression Scale	Geriatric Depression Scale^a^
5. Nutritional status	Subjective Global Assessment or SNAQ^a^	Mini-Nutritional Assessment	(anamnesis by dietician)^a^
6. Comorbidity	Charlson Comorbidity Index^a^	CIRS-G	(anamnesis)^a^
7. Quality of Life^a^	RAND-36^a^	EuroQol-5	EuroQol-5D^a^ Visual Analogue Scale^a^
8. Frailty	Fried frailty indicator^a^	Groningen Frailty Index^a^ Fried Frailty Index (includes 4 m walking test and Handgrip strength)	Rockwood Clinical Frailty score ^c^
9. Caregiver burden	EDIZ-plus^a^	EDIZ^a^	([hetro]anamnesis by social worker) ^a^
10. Estimation of nephrologist		VAS: overall condition Surprise question	Surprise question ^c^
11. Other	Cantril’s ladder, Pain score, Anxiety score Illness perceptions questionnaire^a^	Additional subjective cognition tests (by caregiver): IDDD ^a^ IQCODE ^a^ Neuro-Psychological Inventory ^a^	Outcome Prioritization Tool (treatment goals) ^a^

*ADL* Activities of daily living; *iADL* Instrumental activities of daily living; *GARS* Groningen Activity Restriction Scale; *15-WVLT* 15-Word Verbal Learning Test, immediate and delayed; *SNAQ* Short Nutritional Assessment Questionnaire; *CIRS-G* Cumulative illness rating scale for geriatrics; *EDIZ* ‘Ervaren Druk door Informele Zorg’ *Self perceived burden from informal care*; *IDDD* Interview of Deterioration in Daily life Dementia; *IQCODE* Informant Questionnaire on COgnitive DEcline.

^a^Reassessed measures at follow up, ^b^ Next to the study measures, two hospitals used additional instruments in routine care practice. St. Antonius hospital: a pre-dialysis decision making trajectory, including a home visit by a social worker and assessment of different domains (Katz, Lawton, MMSE, sometimes depression score (GDS), receiving care and living situation). Maasstad hospital: patients ≥70 years, if considered frail by nephrologist, are seen in a separate appointment with a nurse practitioner and assessed with multiple instruments (including: Katz ADL, Lawton iADL, Mini Mental State Examination, Geriatric Depression Scale, Charlson Comorbidity Index, Groningen Frailty Indicator, Timed up and go, Hand grip strength, fall risk, caregiver burden, wellbeing measurement) outcomes are discussed in MDTM and with the patient. ^c^Measures assessed at each visit to the outpatient clinic

Patients were purposively sampled when meeting the inclusion criteria: i.e. aged ≥65 years, chronic kidney disease (CKD; stages 4/5/5D or recent transplantation, eGFR < 20 ml/min/1.73m^2^), and experienced with either one of above mentioned geriatric assessment practices. We invited patients who had positive as well as negative experiences with NGA and with different (future) choices of treatment modality. Patients were asked by phone or face-to-face to participate by the (principal) investigator or treating nurse practitioner, and subsequently sent an information letter and informed consent form per mail. The letter included an invitation for a caregiver to participate, in case patients were unable or unwilling to come to the site on their own. Professionals were recruited per face-to-face, phone or e-mail through a combination of purposive and snowball sampling, if meeting the inclusion criteria: i.e. medical profession involved in providing care for older CKD patients, and involved in one of above mentioned geriatric assessment practices. We purposively invited professionals from various disciplines (i.e. nephrologists, geriatricians, nurse practitioners, dialysis nurses, social workers, dieticians) to ensure representation of all disciplines in geriatric nephrological care. Professionals received an information letter on study purpose, procedure, and confidentiality. Participants were excluded if they were not available at the date of the focus group or unable to get to the interview site. In total, four patients and five professionals declined their invitation, another two patients and two professionals did not show up at the day of the focus groups. Main reasons for decline were inability to attend or for personal reasons. Informed consent was given before start of the focus groups. The Consolidated Criteria for Reporting Qualitative Studies (COREQ) checklist was used as reference for reporting.

### Data collection

Researchers (MB, NB, CV) developed a semi-structured interview topic guide (Additional file 1) which was informed by literature [3, 4, 6, 14, 15, 19] and clinical experiences, and reviewed by nephrologists and geriatricians (HJ, WB, CG, SM). Guiding questions were designed to prompt for views on:

1. Perceptions on the value of geriatric assessment in routine practice
2. Barriers and facilitators to the implementation of geriatric assessment in routine practice

All groups were led in Dutch language by a nurse practitioner (NB) with 40 years’ experience in clinical nephrology and 7 years in geriatric nephrology. It was ensured that none of her own patients were included in groups. The interviewer was trained to conduct focus groups. Each group was held in a quiet and comfortable hospital meeting room, and lasted for a maximum of 3 h, including half an hour break. The interviewer began by clarifying reasons for doing the interviews (i.e. research to develop a nephrology-tailored routine geriatric assessment), explaining confidentiality, introducing herself and observers, and asking participants to introduce themselves. Participants were encouraged to speak freely and discuss their shared opinions as well as differences. One observer (CV) experienced in qualitative research took notes of nonverbal communication and group dynamics. At patient-groups, another observer (NG, RK, KC) was present to provide background information on geriatric assessment practices or feedback to the interviewer.

### Data analysis

Focus groups were audio-recorded, transcribed verbatim, and analysed using inductive thematic analysis, which is a widely used descriptive approach for identifying, analysing and reporting patterns (themes) in qualitative data [20]. A brief summary of discussed topics was given to participants at the end of the focus group, transcripts were not returned for comments or correction. Initial coding and analysis was done by C.V. and N.B. separately, followed by a discussion of meaning of quotes, to confirm and agree on initial codes and (sub)themes. Consistency of the code scheme was checked by M.B. and Y.M. after which some changes were made. Further analysis was done by C.V in close collaboration with N.B. No qualitative research software was used, although coding, memo’s and analyses were documented digitally. Interpretations were iteratively reviewed and critically discussed until consensus was reached within the multidisciplinary research team. Data saturation was assessed by the research team at different stages of analysis, after conducting the groups, coding and analysis, until agreement was reached on sufficiency of information power and no new themes emerged [20–22]. The research team consisted of different backgrounds (i.e. nurse practitioner, health scientist, nephrologists, psychologist, and geriatrician), of whom most were experienced in conducting geriatric assessment in nephrology (except for CV and YM). Selected illustrative quotes were translated from Dutch to English by a native speaker (NB), using back-translation.

## Results

Six focus groups were conducted (*n* = 47, mean 7.8, range 7–9) from November 2017 to January 2018 until data saturation was reached (i.e., until no new information was obtained, or new themes emerged). Three groups concerned the patient-perspective (*n* = 22; i.e. 18 patients [aged 67–88 years] and four caregivers) and three were held with healthcare professionals (*n* = 25). Participant characteristics are shown in Table 2. We identified four themes: (i) perceived characteristics of the older (pre)ESKD patient group, (ii) experiences with geriatric assessment, (iii) barriers and facilitators for implementation of NGA, and (iv) desired characteristics of a suitable geriatric assessment. Illustrative quotations are provided in the results below and additional quotations are shown in Additional file 2.

**Table 2:** Participant characteristics

	Patients (*N* = 18)	Caregivers (*N* = 4)	Professionals (*N* = 25)
Age, mean (range)	79 (67–88)	60 (51–76)	48 (29–61)
Sex, male	9 (50%)	0 (0%)	4 (16%)
Children, yes	17 (94%)		
Civil status			
married/living together with partner	10 (56%)		
widow/no partner	8 (45%)		
Living situation			
independent	12 (67%)		
independent with care facilities (e.g. care at home, alarm bell, or assistance in housework)	6 (33%)		
Education level			
Primary or secondary education	6 (34%)		
Secondary vocational education	5 (28%)		
Higher professional/ university education	7 (39%)		
Treatment status			
Haemodialysis / peritoneal dialysis, n (months since start)	5 (2–21, mean 11.6)		
Transplantation, n (months since transplantation)	3 (4–32; mean 15.3)		
CKD stage 4/5 not on KRT, n	10		
Future choice (if in CKD stage 4/5, not on KRT)			
Haemodialysis / peritoneal dialysis	3		
Transplantation	2		
Conservative kidney management	2		
Multiple modalities open or no decision made	3		
Time since last geriatric assessment, in months, median (range)	5.5 (0.6–14.3)^a^		
Experience in care for 65+ CKD patients, in years, median (range)			5.5 (0.5–28.3)
Clinical role			
Nephrologist			7 (28%)
Geriatrician			4 (16%)
Nephrologist/geriatrician			2 (8%)
Nurse practitioner			2 (8%)
Nurse (nephrology)			3 (12%)
Nurse (other)			2 (8%)
Social worker			4 (16%)
Dietician			1 (4%)
Initiative and medical centre			
COPE study	7 (39%)	–	9 (36%)
Haga hospital, The Hague	4		5
Jeroen Bosch hospital, Den Bosch	–		1
Leiden University Medical Centre	3		2
Reinier de Graaf hospital, Delft	–		1
GOLD study	6 (33%)	1 (25%)	8 (32%)
Academic Medical Centre, Amsterdam	1		–
Gelderse Vallei Hospital, Ede	1		–
Leiden University Medical Centre	1		–
Maasstad Hospital, Rotterdam	–		1
St. Antonius Hospital, Nieuwegein	–		2
University Medical Centre, Utrecht	3		5
Routine care pathway	5 (28%)	3 (75%)	8 (32%)
University Medical Centre Groningen	5	3	8

Abbreviations: *CKD* Chronic kidney disease; *KRT* Kidney replacement therapy. ^a^ Time since last geriatric assessment was substantially longer for patients from the GOLD study (median 11.8 months) versus other initiatives (median 2.2. months), because we reported the time since initial formal geriatric assessment. Additionally, patients had a follow-up after 6 months by telephone

### Perceived characteristics of the older patient group approaching ESKD

Patients and professionals recognized that becoming older is often accompanied by increasing vulnerability and co-morbidity, including polypharmacy and reduced cognition. Both believed that older (pre)ESKD patients need additional support in various domains.

*“In older patients, problems arise that do not occur in younger patients. In many cases, cognitive impairment and life goals also change when life expectancy is shortened. So this does, I think, require extra attention” (Geriatrician, woman 35-40 years)*

*“You grow old, so things go wrong here and there. Yes, that should be taken into account” (Woman 80-85 years, on peritoneal dialysis)*

Patients described that their goals in life may be different in comparison to younger patients (e.g. more focus on independence, care for a sick partner or the ability to spend time with their grandchildren) and their treatment options as more limited, whereas impact of dialysis on daily life may be less invasive.

*“Being on dialysis three days a week for four hours, that is much more invasive for a young patient than for an old patient.” (Woman 80-85 years, on haemodialysis)*

Professionals observed that older patients more often suffer from loneliness and grief compared to younger patients, and had impaired ability to process information due to reduced functional and cognitive functioning. However, they stressed that above mentioned factors are not only related to age but also vary according to individual fitness and social environment. Multiple professionals reported that they wrongly presume to have comprehensive knowledge of their nephrology patients due to the chronic care pathway, and that they realize that without NGA certain geriatric impairments remain underrecognized.

*“As doctors we tend to concentrate on somatic aspects, patient vigour and medical history. We have too little time to explore things like cognition, quality of life, and expectations […] Perhaps we should do things differently.” (Nephrologist, man, 45-50 years)*

### Experiences of using a geriatric assessment

#### (Providers’ perceptions of) patients’ experiences with a geriatric assessment

Patients appreciated the attention during geriatric assessment for multiple aspects of health and daily functioning. They particularly valued the (extra) time and attention they received from professionals. Consequently, patients were able to share more fears and concerns about treatment choices.

*“It was an eye-opener for me. Fortunately, and much to my relief, I discovered that, in spite of my age, I’m able to do things that many peers are not able to do anymore.” (Man 80-85 years, on haemodialysis)*

“*I do notice that when they [patients] are at the geriatrician, they dare to admit more honestly what their fears are. They do not dare to share these fears with a nephrologist because they think that he/she might not want to treat them anymore. With involvement of a very different person or function [the geriatrician] quite different things sometimes come to the surface.” (Nephrologist-geriatrician, woman 40-45 years) “Or the other way around: people who do not want a treatment [..] who almost do not dare to say to me [nephrologist] that they are afraid that they will undermine my role when refusing the treatment”. (Nephrologist, woman 60-65 years)*

Patients experienced the NGA as comparable to a school examination or brain games, either having a strong positive or negative association: some patients felt positively challenged because of the competition with themselves while others were apprehensive. Professionals believed that some patients also experienced tests and subsequent outcomes as confrontational.

*“You want to perform well [patient 5 and 6 mumble in consent]. It is a kind of exam, you do want to get a good grade.” (Woman 70-75 years, transplanted) “Yes, obviously you want to do well.” (Woman 75-80 years, pre-dialysis phase)*

*“If a patient himself thinks he functions good and then he realizes that he does not actually perform well, it can be incredibly confrontational for that patient.” (Nurse practitioner, woman 60-65 years)*

Potential benefits of a repetitive NGA are recognised among most patients, except when treatment was already started, if they were still active in working life, or when cognitive or functional limitations were experienced shortly after a surgery. Interestingly, even the few patients who were negative about testing and to whom the value of doing these tests was unclear, acknowledged the potential of repeating measures to see any progress or decline and to deploy targeted interventions.

*“So you do not understand the purpose [of doing the test]?” (Interviewer). “No, not at all” (Woman 80-85 years, on peritoneal dialysis).“Well, I do! They look at your condition. And after a year, one can off course notice any decline. That is the purpose!” (Man 70-75 years, transplanted after haemodialysis). “I would like to know: are the test-results shared with the treating nephrologist? That should be the case, so they can respond to it – in spheres of social and daily life – and provide more support. Make sure that support is there.” (Woman 80-85 years, on peritoneal dialysis).*

#### Professionals’ experiences with a geriatric assessment

Professionals also considered repeating NGA important; not only to detect changes in patients’ medical situation, but also the social situation. NGA is perceived to be a very important tool to enhance their knowledge of the patient and to identify trends of functioning on geriatric domains over time. Especially cognitive problems and frailty are identified more often when using NGA.

*“And the blind spot: [..] On the ward we had two patients who had just undergone AV-fistula placement, and both turned out to have dementia. In one patient, the diagnosis was missed due to a language barrier. The other was very well educated and was able to camouflage difficulties. Both cases developed a serious delirium after the operation [for which geriatric follow-up was indicated] [..] And that’s when I thought: okay, yes, cognition is indeed something that I don’t specifically look for. So there are other domains that need to be appraised” (Nephrologist-geriatrician, woman 35-40 years)*

Measuring these geriatric domains initiates dialogue on treatment decision and -goals, and provides reason to set targeted interventions. According to most professionals, NGA improves awareness among the whole treatment team to (re)consider different treatment options like conservative kidney management and home dialysis. Notably, caregivers also accredited the importance of NGA in decision making, for safety and competence reasons.

*“Those tests that were done [..] we saw these as very positive, enabling you to make a better choice [..]. If I had memory problems that could cause me to make mistakes, then [test-results] could support me in making a more suitable choice or make a different decision. Your choice becomes more subtle.” (Daughter of male 80-85 years pre-dialysis patient)*

Professionals acknowledged that they themselves should be cautious of labelling patients based merely on test scores. Also, difficult dilemmas may arise when a patient is unexpectedly diagnosed with dementia without a formal request to do so, or when a patient appears to be unable to decide on his/her own treatment. Professionals believed that more evidence is needed on geriatric outcomes after initiating kidney replacement therapy.

*“There is a scientific gap on the course of physical functioning and the course of cognition after start of dialysis therapy in older patients. It has not yet been well-researched. So yes, you cannot inform patients properly either.” (Nephrologist, woman 30-35 years)*

### Barriers and facilitators for implementation of geriatric assessment in routine care

Barriers:

#### Lack of communication about goals and interpretation of tests

A clear explanation of the NGA’ purpose and outcomes is important for patients. To some the tests were unexpected and they felt unprepared whilst other patients recognised benefits of being unprepared.

*“Undoubtedly there may be good reasons to confront patients unexpectedly with a test, but I know my father experienced it as being somewhat unpleasant.” (Daughter of male 80-85 years pre-dialysis patient)*

Furthermore, patients mentioned that they did value discussing personal NGA-results and implications, but that in some hospitals feedback on NGA-results was lacking. Shortcomings in communication about the purpose of NGA in routine care were acknowledged by some professionals. Possible explanations were mentioned, including a sensitive topic and attempts not to worry patients beforehand.

#### Burden for patients

Professionals mentioned that a multi-hour extensive set was more burdensome for frail patients and patients on dialysis, and that in some cases the expected burden was a reason to refrain from testing. A few patients described their tiredness after doing tests for hours. Extra hospital visits were also mentioned by patients as a barrier, especially for those who depend on family for transportation.

#### Masked illiteracy

Professionals warned that masked illiteracy may affect interpretation of test-results of patients with a low education level or a non-Dutch background.

#### Lack of time and budget

Most professionals mentioned that a lack of time was a barrier for implementation of routine NGA. Sufficient time – in duration and consult frequency – is needed to get to know patients and their preferences, and to collect and provide sufficient information for a treatment decision.

In relation to time, professionals perceived limited budget as a barrier, despite that some believed that NGA would potentially save future dialysis treatment costs.

*“Apart from better patient-centred care, it will be more cost effective in my view. When a CGA is performed, you are better informed on the patient’s choice, and this could result in a less expensive treatment choice.” (Nephrologist-geriatrician, woman 40-45 years)*

#### Resistance to involve geriatrics

Both geriatricians and nephrologists reported that nephrologists may feel resistance to consult a geriatrician, partly due to nephrologists’ lack of knowledge on added value of geriatric care.

*“I think that part of the problem is due to the feeling of having to hand over one’s position as leading physician, this feeling that one is not capable of doing this oneself. Perhaps it was more a question of not knowing exactly what the added value of the geriatrician could be.” (Geriatrician, woman 25-30 years)*

#### Loss of knowledge

Professionals reported that, due to a high turnover of young doctors and other team changes, teams often face a loss of knowledge about geriatric nephrology, hindering general awareness for geriatric problems.

Facilitators:

#### Conducting geriatric assessment: trained nurses and home visits

Professionals reported positive experiences when tests were conducted by a trained (specialized) nurse (practitioner) from either the geriatrics or the nephrology department. Additional tests were often conducted by a dietician, social worker or occupational therapist. Professionals believed they played an important role in providing valuable insights into the patients’ situation, for example by conducting home visits.

*“You see a lot during a home visit, for example: you observe the interaction between husband and wife, and between parents and concerned children. When standing at the door, you can already estimate how long you will have to wait. Do you get a cup of coffee or is someone actually up to nothing? These are very subtle signs and observations.” (Social worker, woman 60-65 years)*

#### Multidisciplinary cooperation: involvement of geriatrics

Cooperation between geriatric- and nephrology departments is indispensable to complete non-somatic aspects of frail patients and to support treatment (decisions). Nephrologists experienced the involvement of a geriatrician as supportive in interpreting results, for example: patients had a ‘second opinion’. Most patients confirmed a positive experience with geriatric consultation referring to the given extra attention and additional information.

*“I had a nice conversation with the geriatrician: I was able to discuss my personal experience, real experiences! And I got a lot of information.” (Woman 70-75 years, pre-dialysis phase)*

*“.. at an earlier stage, doubts [about suitability for RRT] have been discussed. That’s just extra support [..] it is a way to share the burden with the geriatrician. It [geriatric impairment] has been objectively tested and that’s the reality. You can then build on that.” (Nephrologist, woman 60-65 years)*

Professionals emphasized that not all older patients need to be referred to a geriatrician and suggested that the NGA could particularly be used to identify patients with (pre)ESKD who may benefit from a geriatric consultation.

#### Multidisciplinary team meetings and reports

Professionals considered a regular multi-disciplinary team meeting (MDTM) of utmost importance to discuss NGA-results and to formulate treatment options.

*“[In a multi-disciplinary team meeting] you can explain how the assessment went, what was striking in conjunction with the numerical results. Sharing this [test-results and striking observations] will give us a better picture, I think.” (Nephrologist, man 45-50 years) “Otherwise, there is a risk that the fragmentation of knowledge will not be brought together into a decision at the multidisciplinary team meeting. Therefore it is very important to have a distinct format with all the aspects of NGA included, and to schedule the discussion of new patients’ test-results and observations.” (Geriatrician, woman 35-40 years)*

Meeting-reports provide useful information for all (future) therapists and general practitioners, and electronic patient records have the potential to facilitate efficient information gathering. However, logistic difficulties were perceived to incorporate all disciplines in the MDTM.

#### Care planning

Professionals reported that care planning could be facilitated by a prolonged first consultation, and combining appointments at multiple disciplines in one hospital visit, immediately followed by a MDTM. Yet, it may be challenging to schedule all appointments in one day.

#### Forerunner and management support

To facilitate multidisciplinary cooperation, professionals reported that it is important to have good informal contact between all disciplines, management support and an enthusiastic forerunner.

### Desired characteristics of a suitable nephrology-tailored geriatric assessment

Patients and professionals described desired characteristics of a NGA suitable for use in practice, relating to the scope and use of test and timing of assessment. Besides, proper execution of tests should include good legibility (reported by patients to lack in instances), and take into account illiterateness and patients’ different cultural backgrounds (mentioned by professionals). No remarkable disparities were observed in assessment preferences for patient groups different in age, gender and educational status.

#### Scope and use of tests

Both patients and professionals stressed the importance to explore beyond the somatic domain, including health-related quality of life, cognitive and social functioning, and other domains such as nutritional status, fall-risk, medication, co-morbidity, mood, home situation and -care, educational level, pain score, life goals and spirituality. Additionally, many professionals indicated that collecting information from caregivers or family members (hetero-anamneses) provides very valuable information.

There was a large variability in how different tests were experienced by patients. For example: mathematics tests were less favoured by patients and sometimes experienced as challenging, whereas others associated them with enjoyable brain games. Professionals favoured specific tests because of familiarity and scientific reasons. For example, among cognitive tests, most professionals agreed that the Montreal Cognitive Assessment (MOCA) was, compared to the Mini-Mental State Examination (MMSE), more appropriate for CKD patients as it would unveil vascular risk. Also, the Stroop Colour and Word Test (i.e. neuropsychological test assessing the ability to inhibit cognitive interference) received little support from professionals. In contrast, clock drawing was considered simple and informative according to most professionals. Patients mentioned recognition of a recurrent Visual Association Test as ‘funny’, and emphasised the need to avoid repetition of tests within and between hospitals.

#### Timing of assessment

To patients ideal testing duration differed from 30 min up to 3 h. The more time the better, applied to some patients. Other patients said they were more concentrated in the morning and preferred to combine appointments for testing with a routine doctor’s consultation. Professionals indicated that it would take between 45 and 90 min to conduct a proper NGA and assess all domains, if necessary divided over disciplines. In addition, several professionals believed that follow-up conversation with the patient to discuss test-results was most important.

*“In my personal opinion, I still think the conversation with the patient matters the most, by far. Usually I’m just not so much into clinimetry but in the long run I do think it is good to have numbers for future comparisons, especially when other doctors become involved.” (Geriatrician, woman 35-40 years)*

Regarding frequency of assessment, most patients said that conducting an NGA once or twice per year is doable. However, opinions varied as some patients would like to take tests frequently while others disagreed. Follow-up measurements were perceived useful by patients to provide insights in decline. But, patients also emphasized that too much repetition of tests within but also between hospitals needs to be avoided.

*“Yes, I do have a bad memory, but I remember the tests. Because in another hospital the same tests were conducted again. Then I thought: Well, they use the same things. [..] and makes it hilarious really, I’m sorry to have to say.” (Woman 81 years, on haemodialysis).*

## Discussion

In this qualitative study, we elicited perspectives about and experiences with hospital-based geriatric assessment in (pre)ESKD patients, by conducting focus group discussions with patients, caregivers and healthcare professionals. We also identified benefits, facilitators and barriers for implementation into routine nephrological care. Although the need for geriatric appraisal for patients approaching ESKD in the hospital setting is widely recognised in guidelines [7, 8] and research [9, 17, 23], the lack of routine assessment practices discloses the need for understanding barriers and preferences of geriatric assessment practices. Our study is the first to explore these important questions using focus group discussions with both patients and professionals.

Our findings show that both patients and healthcare professionals have predominantly positive attitudes about use of a NGA in clinical care of older (pre)ESKD patients. Information provided by NGA creates the much-needed awareness [4] of cognitive and social problems among the whole treatment team, hereby shifting their focus from mainly somatic problems to multidimensional issues. Consistent with previous qualitative studies [14, 16], professionals reported being unaware of deficits in geriatric domains in some patients and that NGA often revealed unidentified cognitive impairments. Nephrologists considered cognitive impairments most important in deciding if a patient is eligible for dialysis [24]. The pivotal role of geriatric assessment in treatment plans and decisions has been reported in oncology [11, 14, 25]. Correspondingly, our study illustrates that discussing NGA-results starts a dialogue about suitability of treatment options between patient and professionals. As a result, patients were able to share more of their fears and concerns. Consistent with a previous study [16], patients reported to highly appreciate the extra time spent on NGA and the attention for impact of CKD on daily functioning. Together with caregivers, patients recognised the benefits of (recurrent) NGA. Outcomes of NGA could enable patients and professionals to assess where supportive interventions may be beneficial. Hence, good multidisciplinary cooperation between nephrology, geriatrics and supportive disciplines is of the utmost importance [26]. Our study illustrates that geriatricians have a complementary function to nephrologists, with the latter knowing the patient and the disease, and the geriatrician having valuable expertise on clinical judgement of frailty and appropriate interventions for older patients.

Despite a growing awareness of the need for geriatric screening among older patients approaching kidney failure [17, 23, 24], implementing NGA in standard care, as recommended in nephrology guidelines [7, 8], remains complicated [9]. Difficulties may relate to lack of a widely-used and trusted test-sets and to implementation hitches. Our study identified several barriers and facilitators for successful implementation of geriatric assessment into routine care. First and foremost, clear communication about the purpose and interpretations of NGA-results with patients is essential. Barriers for interpretation of NGA-results include legibility and masked illiteracy. Since assessment is more burdensome for frail patients [9, 16], an individual approach is recommended. This requires allocated time to perform tests, to interpret test-results and to cautiously present results without overwhelming ill and worried patients [27]. Insufficient time was mentioned as important barrier in our and previous studies [16, 26, 28]. Our findings suggest that between 45 min and 1,5 h is needed, and should be feasible to accomplish a complete picture.

Second, for selecting the right test set*,* although a full CGA remains the gold standard approach [29], our study confirms that adaptation of CGA into a smaller set is necessary and feasible to reduce both provider and patients’ burden [9]. Our study provides some arguments in favour of using specific instruments in nephrology (e.g. Montreal Cognitive Assessment) but there was no outspoken preference for a particular set. Our participants agreed that caregivers provide important information on the patients’ geriatric domains facilitating to provide additional insights in the patients’ situation [14].

Third, barriers for successful interdepartmental cooperation [26, 30] were identified, including: limited availability of geriatricians [9], sparse knowledge and use of geriatric tools among nephrologists [9], and a tension among nephrologists to lose their span of control. Hall et al. [14] have previously described two models of conducting NGA: by a geriatric-trained and experienced nephrologist/nurse practitioner, or by partnership between geriatric medicine and nephrology. Our study supported their findings that both models may work, if tests are conducted by trained and experienced professionals.

Fourth, our findings also highlight the importance of a multidisciplinary team meeting. In oncology, MDTMs led to significant changes in diagnostic findings and altered management plans for more than 10% of cancer patients, including increased likelihood of appropriate staging [31]. More general, (multidisciplinary) geriatric evaluation has led to a primarily less intensive treatment plan in a third of oncology patients, recommendations of non-oncologic interventions in 72% of the patients [11], and, although evidence is sparse, suggests an improvement in clinical outcomes [11, 31]. Finally, sufficient management support and budget was found vital to facilitate resources and to actively support clinicians to efficiently report findings [32, 33].

Strength of our study included the purposive sampling of patients and professionals across (multiple hospitals linked to) three different initiatives performing geriatric assessment in nephrology care. Although one might argue that the experiences with the different practices may have had an impact on our results, it was not our aim to compare the perspectives between the three practices. We rather focussed on the overall perspectives about geriatric assessment among patients approaching ESKD, towards our ultimate goal to develop a generic routine geriatric assessment. Any striking differences between practices were highlighted throughout the results. However, our findings should be interpreted in the light of known limitations related to generalizability and potential for bias. Patients who gave informed consent may potentially be relatively healthier and have a more positive attitude towards their healthcare and NGA. Moreover, patients who lack decisional capacity were not included. Recall bias might have been an issue for some patients since time had passed since they underwent the assessment. A final limitation is research reflectivity: although the whole research team with different backgrounds have judged the results, we cannot rule out that interpretation of the data is coloured by theoretical preconceptions and a pre-set aim and involvement (except for YM) to implement NGA in routine care.

Further quantitative research is needed on the clinical- and cost-effectiveness [10] of NGA, and the beneficial effects on treatment decision making and supportive interventions, as well as on the feasibility and acceptability of implementation of NGA in routine care [32]. This may give insight into how new diagnoses of cognitive defects and frailty lead to changes in treatment plans and avoidance of hospitalization, and on the necessity of test repetition. Furthermore, consensus on a tool set and long-term systematic collection of data may enhance clinical- and research comparability, enabling individual predictions in disease progression. We hope that our lessons and recommendation described (please also see Table 3) help to successfully integrate NGA into regular clinical care.

**Table 3:** Lessons learned and considerations for NGA implementing into routine clinical practice

**Patients’ involvement and potential benefits** • Clear communication of goals and outcomes (and interpretation) of tests • Consider the burden for patients individually, pay attention to results that might be confrontational • Spending 45–90 min in total to complete the NGA is acceptable and feasible • Repeated measurements to assess progression and to identify the need for additional supportive interventions • Caregivers can provide important information about patients’ situation at home	
**Selecting the right test set** • Legibility and masked illiteracy can be important barriers • Adaptation of CGA into a smaller set for nephrology (NGA) is possible and feasible • Different instruments are suitable for usage in routine clinical practice, as long as all geriatric domains (i.e. somatic, social, functional and cognitive) are covered • Uniform implementation of NGA is necessary in response to the need for scientific evidence of geriatric measures on outcomes to predict individual progress of the disease.	
**Sufficient expertise and multidisciplinary cooperation** • Collaboration between nephrology and geriatrics departments is of utmost importance to provide complementary patient care • Multidisciplinary team meetings are key to success • Assessment of geriatric domains can be done by either the nephrology and geriatrics department, once professionals are trained and experienced • Barriers for successful cooperation between the geriatric and nephrology department include: apprehension among nephrologists about loss of span of control, sparse knowledge and use of geriatric tools, and a high turnover of doctors	
**Supporting structures** • Sufficient management support providing essential resources (e.g. time and money) for innovations • Securing the appropriate workforce, especially the availability of geriatricians • Value of NGA should be proven, resulting in evidence and directive guidelines	

## Conclusions

In conclusion, we observed that both patients and professionals experienced a great potential for hospital-based standardized geriatric assessment to improve (pre)ESKD patient care and clinical outcomes by identifying, discussing and targeting multidimensional problems. Implementation into routine care seems promising and achievable, once barriers and facilitators are recognized, but future studies are needed to investigate the effects of implementing geriatric assessment.

## Supplementary Information

**Additional file 1.** Semi-structured interview topic guide. Semi-structured interview topic guide (questionnaire) developed for this research and used in the focus group discussions.

**Additional file 2.** Additional illustrative quotations. Additional illustrative quotations by theme and subtheme.

## Data Availability

Transcripts of focus group interviews are stored in electronic format on secure servers at the Leiden University Medical Centre. All data generated during analysis in the study are also stored on these servers; where considered necessary for publication, they have been included in this published article. For privacy reasons, data will not be shared publicly.

## Abbreviations

CGA: Comprehensive geriatric assessment.
CKD: Chronic kidney disease.
ESKD: End-stage kidney disease.
NGA: Nephrology-tailored geriatric assessment.
MDTM: Multidisciplinary team meeting.
