# Supplementary material for: Perspectives and experiences of patients and healthcare professionals with geriatric assessment in chronic kidney disease: a qualitative study
Source: BMC Nephrol. 2021 Jan 6;22:9. doi: 10.1186/s12882-020-02206-9 (PMC7789317; doi:10.1186/s12882-020-02206-9)
Supplement: Supplementary file 1 — Additional file 1. Semi-structured interview topic guide. Semi-structured interview topic guide (questionnaire) developed for this research and used in the focus group discussions. [file 12882_2020_2206_MOESM1_ESM.docx]

Additional file 1: Semi-structured interview topic guide

*Welcome (purpose and procedure of focus group discussion, confidentiality, introducing)*

1. **How does care differ for patients approaching ESKD above 65 years of age, compared to patients of a younger age?**

Probe for aspects missing in pre-ESKD care of older patients.

1. **What does a patient [and professional] need in order to enhance the treatment decision making process?**

Write down a number of points, please explain.

1. **How did you experience doing the geriatric tests [from patient and professional perspective]?**

Probe for: factors of patient-impact and feasibility (frequency, setting, caretakers, way of assessing, specific tests), psychological experience or burden

1. **To what extent do you think geriatric care (i.e. geriatric tests, consultation and advice of geriatrician) contributed to the regular nephrology care?**

Probe for: collaboration between nephrology and geriatric department, contact with the geriatrician, impact of the tests, including effect on renal replacement therapy (RRT) choice.

1. **Which factors play an important role for older patients in pre-ESKD care and decision making for RRT?**

Probe for: factors in geriatric domains (social, psychological, somatically, functional), role of partner and family, other influences and crucial aspects in decision making.

1. **To what extent did the results of the geriatric assessment influence treatment choice (RRT) [from a patient and professional view]?**

Probe for: experience with specific tests

1. **Which barriers would you expect if a geriatric assessment is introduced in routine care of older patients with pre-ESKD? Which facilitators could you foresee? Please write down three main barriers and three main facilitators.**

Probe for: general aspects (psychological, social, organizational barriers and facilitators) and specific aspects (intrinsic/extrinsic psychological barriers and facilitators)

1. **If you had the chance to design your own program to screen older patients with ESKD, which tests would you definitely include?**

Probe for: reasons for specific preferences, practical issues, patient-related factors, what is needed for successful implementation.

1. **Did you miss a topic in this conversation on nephro-geriatric care? Is there anything you would like to add?**

Questions were asked to both patients and professionals, with minor adjustments in form of address for the group of interest. Parts between squared brackets were only used in the interview protocol for professionals. The interview guide was not pilot tested but minor changes were conducted after each focus group.
