# Supplementary material for: Perspectives and experiences of patients and healthcare professionals with geriatric assessment in chronic kidney disease: a qualitative study
Source: BMC Nephrol. 2021 Jan 6;22:9. doi: 10.1186/s12882-020-02206-9 (PMC7789317; doi:10.1186/s12882-020-02206-9)
Supplement: Supplementary file 2 — Additional file 2. Additional illustrative quotations. Additional illustrative quotations by theme and subtheme. [file 12882_2020_2206_MOESM2_ESM.docx]

Additional file 2: Additional illustrative quotations

**Theme 1: Characterisations of the older ESKD patient group**

- *“What I often hear from older people, is that* **–** *when becoming older* **–** *they have to deal much more frequently with bereavement. Relatives they have lost and the inherent loneliness which follows.” (Social worker, woman 30-33 years)*
- *“In my view, older patients are often more settled in life. The older patients over 70 years of age who visit us are sometimes fitter compared to the 40-50 year old’s who have all kinds of cardio-vascular problems and diabetes. [..] I find it difficult to generalize and to put a label on all older patients, since it depends very much on each individual” (Nephrologist, man 45-50 years)*
- *“Some nephrology patients we know for over 10 years [..] and some of them are fit. But I think that, particularly in the field of cognition, we do not look close enough.” (Nephrologist, woman 30-35 years)*

**Theme 2: Experiences of use of a geriatric assessment**

**Experiences**

- *“I am very positive about the tests, because I want to know about my situation. When you get tested, you know where you stand. I like that. It’s nice when they ask me as many questions as possible about things that I can still do.” (Man 85-90 years in pre-dialysis phase)*
- *“If your test-results are positive, it gives you a boost.” (Woman 70-75 years transplanted)
  “Yes, it gives you courage. Yes, it is really encouraging.” (Woman 75-80 years, on haemodialysis; in response)*

**Benefits**

- *“Geriatricians are especially good at interpreting the CGA results of comprehensive geriatric assessment [especially the* comprehensive *aspect of the assessment] and generating a treatment plan to ensure that dialysis goes as well as possible” (Geriatrician, woman, 45-50 years)*

**Concerns**

- *“The thing I find difficult [..] is when patients themselves do not make the medical request because of memory problems, but when I myself suspect dementia. So it is not the reason for their visit, not their own requisition. That’s sometimes a difficult dilemma, what do you do in that case?” (Geriatrician, man 45-50 years)*

**Theme 3: Potential barriers and facilitators to implementation of geriatric assessment**

*Barrier: communication on goals and interpretation of tests*

- *“People are very sensitive if, based on their age or functional impairments alone, they are discounted up front and feel unworthy.” (Social worker, woman 60-65 years)*

*Barrier: burden for patients*

- *“Most patients [on dialysis] do not want to continue [geriatric assessment] anymore. They think it is a hassle and they really say it is too burdensome. They really do not want to stay at the dialysis department for a minute longer than the dialysis itself.” (Dialysis nurse, woman 40-45 years)*
- *“So, you must take into consideration that it should not become an extensive test battery, that is too time consuming and therefore unsuitable in the outpatient clinic.” (Nephrologist, woman 35-40 years)*

*Barrier: masked illiteracy*

- *“I sometimes wonder whether we should screen for illiteracy, since this is a neglected problem.” (Nephrologist-geriatrician, woman 40-45 years)
  “Yes, patients are very well able to mask that…[illiteracy].” (Dialysis nurse, woman 40-45 years)
  “They are well able to partake in discussions, so you overestimate them and the information you provide – and think you deliver well – just isn`t comprehended.” (Nephrologist-geriatrician, woman 40-45 years; again)*

*Barrier: Resistance to involve geriatrics*

- *“There is probably also some tension – at least that is what I notice in my hospital – between the specialists internal medicine and geriatricians. That they would prefer to keep those geriatricians out.” (Nephrologist, woman 35-40 years)*

*Barrier: loss of knowledge*

- *“I see one other obstacle [..] that has to do with knowledge and transferability of information. Frequently, fellows [nephrologists in training] perform the consultations, however we [nephrologists] may fail to properly inform them about the procedures and protocols involving these [older] patients. Consequently, the fellows will do it in their own way. So I think that is a learning point for us.” (Nurse practitioner, woman 50-55 years)*

*Facilitator: Care planning*

- *“In our practice, patients 70 years and older are triaged into our nephrology-geriatric care pathway and therefore receive more consultation time. [..] One hour is allocated for first consultations with patients 70 year of age or older, and routine visits are allocated half an hour.” (Nurse practitioner, woman 50-55 years)*

**Theme 4: Desired characteristics of a suitable nephrology-tailored geriatric assessment**

- *“For me as often as possible. So at every visit. I think that is fine.” (Man 75-80 years pre-dialysis phase)*
- *“I would find it terrible to have to do a repeat of the tests: an inspection every year.” (Woman 80-85 years on haemodialysis)*
